# Persistent Corneal Decompensation due to Anterior Dislocation of Soemmering Ring Cataract

**DOI:** 10.1155/2017/4672107

**Published:** 2017-11-19

**Authors:** Travis Peck, Shruti Aggarwal, Sandra Johnson

**Affiliations:** ^1^Department of Ophthalmology, University of Virginia, Charlottesville, VA, USA; ^2^Department of Medicine, Reading Hospital, Reading, PA, USA

## Abstract

**Purpose:**

We present a case of a patient with Soemmering ring after cataract surgery and a potential complication that can arise as a result of its presence.

**Observations:**

A patient with history of ruptured globe status after repair and lensectomy, complicated by aphakic secondary open angle glaucoma, was referred for management of second injury to the same eye. This injury resulted in Soemmering ring dislocation into the anterior chamber. The cortical material caused a significant increase in intraocular pressure and corneal decompensation. Surgical removal of the Soemmering ring and Ahmed glaucoma tube implant was performed with control of intraocular pressures; however corneal edema could not be reversed.

**Conclusions and Importance:**

This case report illustrates the serious consequences that can be caused by Soemmering rings without early surgical intervention. Care must be taken to completely remove cortical material during cataract surgery to prevent their formation.

## 1. Introduction

A Soemmering ring is a form of postcataract opacity occurring after the central section of the lens has been removed secondary to trauma or surgery. It is caused by adherence between the residual outer part of the anterior capsule and the posterior capsule. This adherence forms a ring around the periphery of the capsule under which lens remnants are trapped. It is proposed that hyaline capsular material is deposited in this ring and that epithelial cells can proliferate, causing the area to thicken [1]. Fortunately, the ring formed is hidden by the iris and is too peripheral to cause visual symptoms.

Soemmering rings most often come to clinical attention when dislocation occurs. The most commonly encountered complication of anterior dislocation is glaucoma while posterior dislocations typically result in visual disturbances [2]. Given the rarity of dislocation, optimal management remains unclear. Here, we report a case of glaucoma and corneal edema secondary to an anteriorly dislocated Soemmering ring.

## 2. Case Report

A 52-year-old male presented to the Emergency Room at the University of Virginia with the chief complaint of blunt trauma to his left eye.

His past ocular history was significant for blunt trauma to the same eye 15 years earlier, resulting in a ruptured globe, traumatic cataract, and traumatic retinal detachment for which he had undergone surgical repair with lensectomy, vitrectomy, and a scleral buckle placement. He was left aphakic after the surgery. His postoperative course was complicated by traumatic glaucoma, for which he was followed regularly at the University of Virginia Department of Ophthalmology for the three years leading up to presentation.

His baseline corrected distance visual acuity (CDVA) prior to the second trauma was 20/20 in the right eye and 20/60 in the left eye. Intraocular pressure (IOP) was 15 mm Hg in the right eye and 18 mm Hg in the left eye. He was noted to be aphakic with a peripheral ring of opacified cortex on slit lamp biomicroscopic examination (SLE). Gonioscopy showed open angles with anterior insertion of the iris root. Ultrasound pachymetry revealed a central corneal thickness (CCT) of 597 microns in the right eye and 620 microns in the left. Humphrey automated visual fields (AVF) 24-2 showed no depression in the right eye and arcuate defects more prominent superiorly than inferiorly in the left eye. Spectral Domain optical coherence tomography of the retinal nerve fiber layer (SD-OCT RNFL) (Cirrus, Carl Zeiss Meditec Inc.) was within normal limits in the right eye (96.13 microns) and revealed diffuse thinning in the left eye with an average thickness of 54.14 microns. The IOP in the left eye was maintained in the teens with a regimen of brimonidine, dorzolamide, timolol, pilocarpine, and latanoprost.

**Figure 1:**
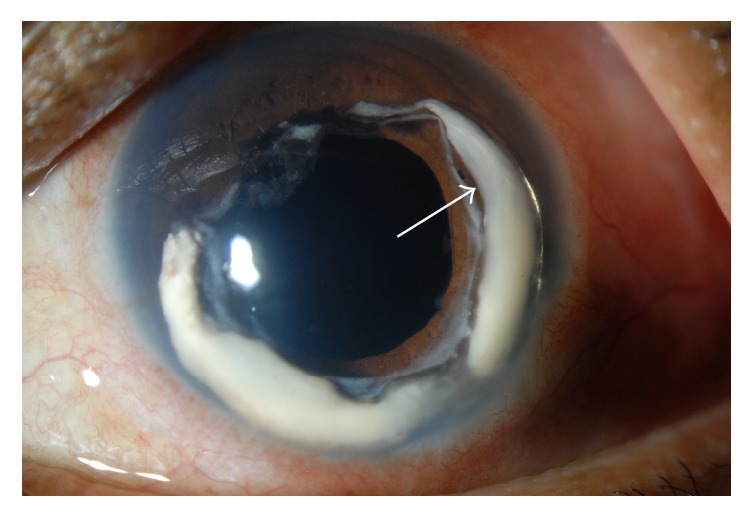
Slit lamp photograph of the right eye showing an incomplete ring of milky white opaque cortical lens material, Soemmering ring, dislocated into the anterior chamber indicated by the arrow.

The patient presented to the Emergency Department with left medial and orbital floor fractures on CT scan of the facial bones. On exam, vision was 20/40 in the right eye and 20/200 in the left eye. IOP was 14 mm Hg in the right and 16 mm Hg in the left. SLE revealed an anterior dislodgement of the previously noted Soemmering ring cataract into the anterior chamber (Figure 1). No corneal touch was noted. AVF and SD-OCT RNFL were stable compared to his previous imaging three years earlier. His subsequent elevation of IOP and inflammation was managed initially with oral acetazolamide and four times daily prednisolone acetate eye drops in addition to his previous glaucoma drops. The patient discontinued using the prescribed prednisolone after one week. IOP was in the high twenties for one month before it stabilized to the low teens. Oral acetazolamide was stopped at this time.

After seeing him weekly for one month, it was elected to space visits out to every three months and to defer surgical intervention given the stability of his exam. However, after some initial improvement, his CDVA continued to worsen throughout this time. One year after the trauma, the Soemmering ring was noted to be touching the cornea for the first time on SLE. Corneal edema was also seen inferiorly around the area of touch. AVF revealed worsening diffuse depression, stable superior arcuate defect, and a new central depression. At this time, it was elected to proceed with surgical intervention. The patient underwent removal of the cortical material from the anterior chamber via paracentesis and scleral tunnel 14 months after the second trauma. Postoperatively, IOPs stayed high despite maximal medical management consisting of brimonidine, dorzolamide-timolol, pilocarpine, latanoprost, and prednisolone acetate. Thus, he underwent Ahmed glaucoma tube implant (New World Medical) surgery in the left eye after which IOP was well-controlled once again in the teens on timolol drops.

The patient has now been followed for two years after glaucoma surgery. While IOP has stayed in the low teens, vision and corneal edema have not improved. The CCT has continued to increase, measuring between 750 and 800 microns over the past six months. On his most recent exam, CDVA was 20/20 on the right and 3/200 in the left. IOP was 14 mm Hg in the right eye and 12 mm Hg in the left. On SLE, he had microcystic corneal edema, significant corneal haze, and scarring, and an in-place shunt tube without any corneal touch. He was given hypertonic saline ointment, prednisolone acetate, and later a bandage contact lens, to help with the resultant pain. He was given the option of Descemet's stripping automated endothelial keratoplasty for definitive management. Penetrating keratoplasty was also offered given his corneal scarring as well as his history of vitrectomy, lensectomy, and high astigmatism. He has elected to defer any surgical intervention as visual prognosis is uncertain.

## 3. Discussion

Modern cataract surgery has a high rate of success without significant postoperative complications. One of the more common potential complications is posterior capsular opacification (PCO). One mechanism behind PCO is the proliferation of lenticular epithelial cells that can remain within the capsule after surgery. A Soemmering ring is a specific type of PCO that is formed when the epithelial cells form a ring around the periphery of the capsular bag. This ring is allowed to form because the anterior capsule leaflet fuses to the posterior capsule and protects the cells and lens fibers from the lytic aqueous humor [1].

There are multiple factors that predisposed this patient to Soemmering ring dislocation. It has been documented that dislocations are associated with myopia. Proposed mechanisms include an atrophied and dysfunctional ciliary body or a decreased viscosity of the aqueous humor [3]. It is likely that this patient was myopic prior to lensectomy as he wore a −1.50 diopter prescription in the unaffected contralateral eye. Younger age at initial injury or lensectomy increases risk of both formation and dislocation of Soemmering rings, presumably due to a higher proliferative potential of remaining epithelial cells. The actual dislocation event typically occurs later in life as zonules weaken [2]. Trauma is an immediate cause for Soemmering ring dislocation and was the primary cause in this case.

There have only been a few published reports of dislocated Soemmering rings in the literature. Anterior dislocation is more common and typically leads to glaucoma [2, 4]. Mechanisms proposed include pupillary block due to an enlarging ring, progressive synechial angle closure without pupillary block, and acute angle closure from enlarged Elschnig pearl (proliferation of epithelial cells along posterior capsule) [4]. Complications of anterior dislocation can also occur with intermittent corneal touch that only occurs with blinking. Posterior dislocation of Soemmering ring has also been documented and resulted in significant visual disturbance in all three cases [2]. Here, we report a case of an anteriorly dislocated Soemmering ring causing worsening corneal edema and open angle glaucoma secondary to inflammation.

The patient in this study went approximately 15 years without any signs of dislocation of the Soemmering ring until blunt trauma to the orbit caused anterior dislocation. Although the patient was being treated for traumatic glaucoma, the lens remnants within the capsule were not causing any symptoms, as is typical for this process [5]. The patient did have mildly compromised corneal endothelial function in the left eye prior to the second trauma as indicated by increased CCT, but once the cortical material dislocated into the anterior chamber, it caused progression of glaucoma as well as significant worsening of corneal edema and visual acuity. After a period of observation, it became clear that the lens remnants required surgical removal. Despite surgery, visual acuity continued to deteriorate and corneal endothelial dysfunction worsened postoperatively. It appears as though irreversible damage was done by the corneal touch that can only be managed with corneal transplant.

This case illustrates the potential morbidity associated with PCO and the ongoing need to focus on reducing its occurrence. Improved surgical techniques along with new lens materials and designs aimed at decreasing PCO formation have resulted in rates of PCO requiring YAG laser treatment falling to 1.22% in 2013 from 33.4% in the early 1990s [6, 7]. Menapace describes success with the technique of posterior optic buttonholing, in which the anterior capsule leaflet is not permitted to contact the optic [8]. This prevents capsular fibrosis and the formation of PCO, specifically Soemmering rings.

Three-piece IOLs have been shown to significantly retard PCO formation compared to one-piece IOLs. Larger optic pieces also reduce PCO. In regard to material, second-generation silicone and hydrophobic acrylic lenses decrease PCO by increasing capsular contraction around the IOL [6]. Dewey postulates that it takes up to five years of cortical regeneration to form a significant Soemmering ring [9]. This process could be inhibited by a square haptic that induces a fibrotic capsular bend and anterior capsular fibrosis, which would restrict the ring to the far periphery of the capsule. Our patient could not benefit from any of these measures to restrict PCO growth as he was left aphakic after lensectomy.

Significant morbidity and loss of visual acuity could have been avoided if this patient's Soemmering ring was removed before it dislocated. However, given the rarity of such an event and the asymptomatic nature of a Soemmering ring within the lens capsule, it does not seem advisable to attempt prophylactic removal of the cortical material. A better final outcome may also have been achieved by removing the dislocated cortex immediately rather than after months of medical management. This case shows that lens fragments in the anterior chamber can be inflammatory and are especially injurious if in contact with the cornea. Additionally, fragments that do not initially touch the cornea can move to become in contact with it, as seen in this case. Thus, optimal management may be immediate lens fragment removal from the anterior chamber before further complications arise.
